# A fluorescent reporter system for anaerobic thermophiles

**DOI:** 10.3389/fbioe.2023.1226889

**Published:** 2023-07-05

**Authors:** Rémi Hocq, Sara Bottone, Arnaud Gautier, Stefan Pflügl

**Affiliations:** ^1^ Institute of Chemical, Environmental and Bioscience Engineering, Technische Universität Wien, Vienna, Austria; ^2^ Christian Doppler Laboratory for Optimized Expression of Carbohydrate-Active Enzymes, Institute of Chemical, Environmental and Bioscience Engineering, Technische Universität Wien, Vienna, Austria; ^3^ Laboratoire des Biomolécules (LBM), Centre National de la Recherche Scientifique (CNRS), Sorbonne Université, École Normale Supérieure, Université PSL, Paris, France; ^4^ Institut Universitaire de France, Paris, France

**Keywords:** anaerobic thermophiles, fluorescent reporter, fluorescence-activating and absorption-shifting tag, genetic tools, acetogen, *Thermoanaerobacter*

## Abstract

Owing to their inherent capacity to make invisible biological processes visible and quantifiable, fluorescent reporter systems have numerous applications in biotechnology. For classical fluorescent protein systems (i.e., GFP and derivatives), chromophore maturation is O_2_-dependent, restricting their applications to aerobic organisms. In this work, we pioneered the use of the oxygen-independent system FAST (Fluorescence Activating and absorption Shifting tag) in the thermophilic anaerobe *Thermoanaerobacter kivui*. We developed a modular cloning system that was used to easily clone a library of FAST expression cassettes in an *E. coli*—*Thermoanaerobacter* shuttle plasmid. FAST-mediated fluorescence was then assessed *in vivo* in *T. kivui*, and we observed bright green and red fluorescence for cells grown at 55°C. Next, we took advantage of this functional reporter system to characterize a set of homologous and heterologous promoters by quantifying gene expression, expanding the *T. kivui* genetic toolbox. Low fluorescence at 66°C (T_opt_ for *T. kivui*) was subsequently investigated at the single-cell level using flow cytometry and attributed to plasmid instability at higher temperatures. Adaptive laboratory evolution circumvented this issue and drastically enhanced fluorescence at 66°C. Whole plasmid sequencing revealed the evolved strain carried functional plasmids truncated at the Gram-positive origin of replication, that could however not be linked to the increased fluorescence displayed by the evolved strain. Collectively, our work demonstrates the applicability of the FAST fluorescent reporter systems to *T. kivui*, paving the way for further applications in thermophilic anaerobes.

## Introduction

Anaerobic microorganisms are key effectors for the sustainable production of biofuels and biochemicals, as they typically display high carbon and energy efficiency as well as substrate versatility required to operate economically viable bioprocesses (Weusthuis et al., 2011). Among the plethora of industrially relevant anaerobes, thermophilic microbes offer additional benefits compared to mesophiles, namely, higher catalytic turnover rates, lower cooling/distillation costs and contamination risks (Turner et al., 2007). Higher operating temperatures also make thermophiles ideal candidates for consolidated bioprocessing, a system combining in a single step all the processes necessary for lignocellulosic biomass conversion to value-added products (Lynd et al., 2005).

The Gram-positive *Thermoanaerobacter kivui* is arguably one such anaerobic thermophile of industrial interest. Its pertinence stems mostly from its capacity to efficiently employ the reductive acetyl-CoA pathway (also known as the Wood-Ljungdahl pathway, WLP) to convert gaseous C1 moieties (CO_2_, CO) into acetate (Hess et al., 2014; Weghoff and Müller, 2016). Autotrophic growth occurs at temperatures between 50°C and 72°C (T_opt_ = 66°C) and is particularly fast (doubling time of around 2 h on H_2_/CO_2_) (Leigh et al., 1981). In addition, fast growth does not require expensive media supplements such as yeast extract, peptone or vitamins. Lastly, *T. kivui* is easily compatible with reverse (Weghoff and Müller, 2016) and forward (Basen et al., 2018; Moon et al., 2019; Jain et al., 2020; Katsyv et al., 2021a; Katsyv et al., 2021b; Jain et al., 2021; Katsyv and Mueller, 2022) genetics approaches, simultaneously boasting fast-evolving capacities (Zeldes et al., 2023) and low genetic barriers (Basen et al., 2018).

Despite their potential, anaerobic thermophiles like *T. kivui* mostly comprise non-model microorganisms and as such suffer from a general lack of genetic tools that hamper metabolic engineering efforts (Lin and Xu, 2013; Crosby et al., 2019). Reporter systems for instance still rely on tedious multi-step enzymatic assays (Bartosiak-Jentys et al., 2012; Dai et al., 2022), although measurements could be greatly facilitated by using fast and direct systems, such as fluorescent reporters. However, classical fluorescent systems have so far been reported to be incompatible with strict thermoanaerobic conditions (Bartosiak-Jentys et al., 2012; Lin and Xu, 2013; Reeve et al., 2016). In fact, until only recently the lack of reliable fluorescent reporters was deemed a significant obstacle for studying classic mesophilic anaerobes (Charubin et al., 2018), as popular fluorescent systems (e.g., GFP and derivative proteins) necessitate an oxygen-dependent maturation step to activate their fluorescence (Heim et al., 1994; Barondeau et al., 2006). A significant breakthrough was achieved by using oxygen-independent systems based on protein interactions with either natural [e.g., Flavins (Cui et al., 2012; Li et al., 2014; Xu et al., 2015; Bruder et al., 2016; Buckley et al., 2016; Molitor et al., 2016; Cho and Lee, 2017; Mordaka and Heap, 2018; Seo et al., 2018)] or synthetic ligands (Streett et al., 2019; Charubin et al., 2020a; Chia et al., 2021). This approach subsequently rendered anaerobes amenable to the wide array of applications fluorescent protein systems enable, including characterization of genetic parts (Streett et al., 2019; Charubin et al., 2020a; Flaiz et al., 2022) or gene expression (Xu et al., 2015; Bruder et al., 2016; Cho and Lee, 2017; Flaiz et al., 2021; Mook et al., 2022), protein and cell imaging via fluorescent microscopy (Cui et al., 2012; Li et al., 2014; Buckley et al., 2016; Seo et al., 2018; Streett et al., 2019; Charubin et al., 2020a; Charubin et al., 2020b), and study of microbial consortia and synthetic cocultures dynamics with flow cytometry (Charubin et al., 2020b; Flaiz et al., 2022).

A number of oxygen-independent fluorescent systems based on ligand-protein interactions have been developed for thermophilic applications (Vettone et al., 2016; Visone et al., 2017; Wingen et al., 2017; Nazarenko et al., 2019; Mattossovich et al., 2020; Merlo et al., 2022). However, only one of those fluorescent systems has been tested *in vivo* (Visone et al., 2017). Fluorescence relies on an engineered thermostable enzyme (O^6^-alkylguanine-DNA-alkyltransferase from *Saccharolobus solfataricus*, H5) that reacts covalently with O^6^-benzyl-guanine (BG) or O^2^-benzyl-cytosine (BC) derivatives, similarly to the SNAP- and CLIP-tag technologies, respectively (Keppler et al., 2003; Gautier et al., 2008). H5 was used in conjunction with a fluorescent BG derivative (BG-FL) in the obligate aerobe *Sulfulobus islandicus* (T_opt_ = 75°C) for fluorescent microscopy imaging but was not tested in an anaerobic thermophile. Although successful, this approach presents some disadvantages that might have hampered its subsequent use, including the need to remove any endogenous O^6^-alkylguanine-DNA-alkyltransferase gene homolog and a relatively tedious staining protocol, resulting from the time of the covalent labeling reaction and the extensive washing necessary to remove unbound BG derivatives.

**FIGURE 1:**
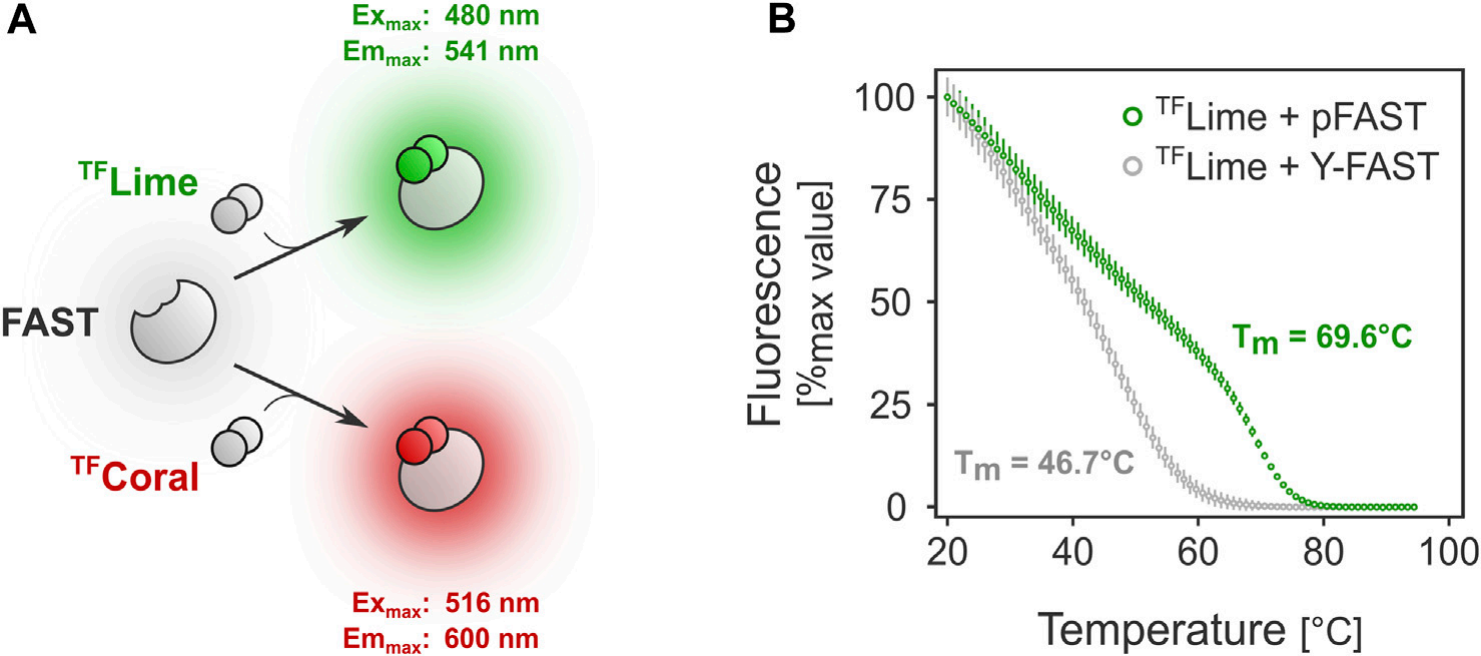
FAST-mediated fluorescence thermostability *in vitro*. **(A)**. FAST relies on ligand-tag binding for fluorescence absorption/emission. **(B)**. Melting temperature determination for two FAST variants: Y-FAST and pFAST in complex with ^TF^Lime (HMBR).

The fluorescence-activating and absorption-shifting tag (FAST) technology relies on the reversible binding of a ligand (so-called “fluorogen”) to a 14 kDa protein tag (Plamont et al., 2016) (Figure 1A). In this system, high contrast fluorescence is emitted only when the fluorogen is bound to the FAST tag and excited at an adapted wavelength, therefore removing the need of extensive washing post-staining. The fluorogen itself penetrates cells extremely fast, which produces quasi-immediate fluorescence upon fluorogen addition. FAST comes with a versatile array of fluorogens and tags, the combination of which forms a comprehensive toolbox suitable for a large array of applications (Plamont et al., 2016; Li et al., 2017; Li et al., 2018; Tebo et al., 2018; Tebo and Gautier, 2019; Li et al., 2020; Benaissa et al., 2021; Tebo et al., 2021). The latest tag variant, pFAST, was engineered from the original tag Y-FAST by directed evolution and displays increased fluorogen binding affinity as well as fluorescence brightness, and is additionally compatible with a collection of fluorogens that span the visible spectrum (Benaissa et al., 2021). In mesophilic acetogens, fluorescent reporter systems based on FAST have been shown to be functional on several occasions (Flaiz et al., 2021; Flaiz et al., 2022; Mook et al., 2022). However, neither the extensive FAST toolbox nor other ligand-protein fluorescent reporter systems have been tested *in vivo* in anaerobic thermophiles.

In this study, we show that pFAST displays a high melting temperature (T_m_) and can serve as a fluorescent marker in the thermophilic anaerobe *T. kivui*. We engineered a modular cloning system based on Golden Gate assembly for this relatively uncommon microbe, which allowed easy construction and introduction of pFAST-bearing shuttle plasmids. pFAST-mediated fluorescence was subsequently established at 55°C and 66°C, which was exploited to characterize promoter parts and to engineer novel Gram-positive origins of replication.

## Results

### FAST tag thermostability *in vitro*

FAST-mediated fluorescence greatly depends on the choice of the FAST tag. Because protein stability can differ significantly among variants, we first investigated the behavior at higher temperatures of Y-FAST and pFAST, the earliest and latest FAST tag versions, respectively (Plamont et al., 2016; Benaissa et al., 2021). Preliminary *in vitro* assays showed that the melting temperature for Y-FAST and pFAST in complex with ^TF^Lime were substantially different (Figure 1B). In the case of pFAST, the observed T_m_ was close to 70 °C (*versus* ≈47°C for Y-FAST), underlining its potential compatibility for use as a fluorescent reporter with bacteria from the *Thermoanaerobacter* genus, which typically display an optimal growth temperature between 60°C and 70°C.

### A Golden gate assembly-based cloning system for *Thermoanaerobacter*

*Thermoanaerobacter kivui* DSM 2030 was chosen as the model system for this study because of its proven track record for forward genetics approaches. *T. kivui* has notably been shown to be naturally competent, and can be transformed with pMU131 derivatives, an *E. coli*–*Thermoanaerobacter* shuttle plasmid (Shaw et al., 2010; Basen et al., 2018). In addition, gene coding for natural homologs of FAST proteins could not be found in *T. kivui* genome, indicating FAST could likely be used without prior modification of the strain.

**FIGURE 2:**
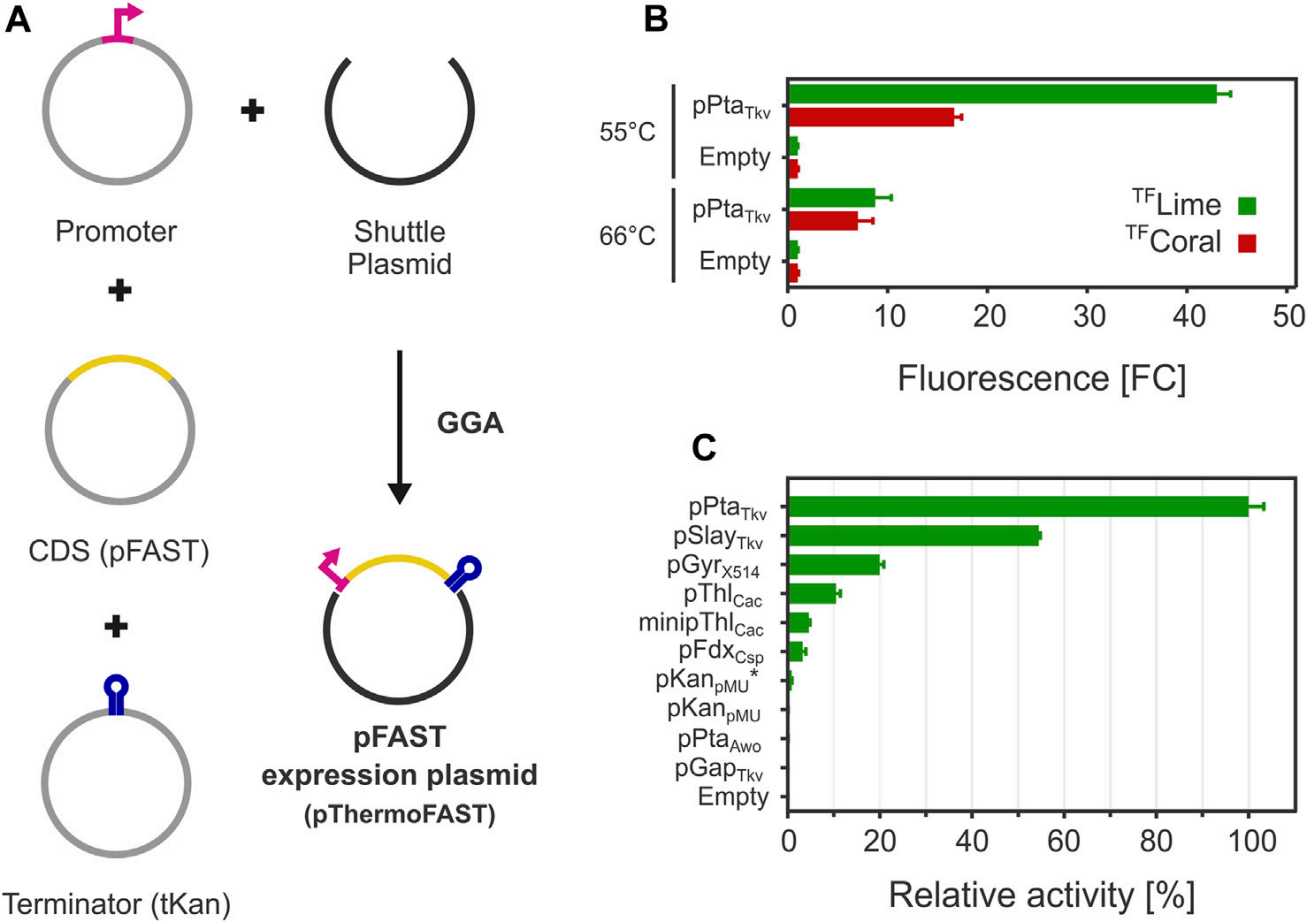
FAST-mediated fluorescence functionality in *T. kivui*. **(A)** A Golden Gate assembly system was engineered for introducing pFAST expression cassettes in *T. kivui*. **(B)**. Fluorescence observed with a plate reader with for cells grown at 55°C and 66°C (5 µM ^TF^Lime or ^TF^Coral, *n* = 3, error bars correspond to the SEM). **(C)**. Fluorescence measured with a plate reader of a library of pFAST expression cassettes with different promoters for cells grown at 55°C (5 µM ^TF^Lime, *n* = 3, error bars correspond to the SEM).

To facilitate the construction of pFAST expression cassettes, we adapted pMU131 to be compatible with a simplified hierarchical Golden Gate assembly system based on GoldenMOCS (Golden Gate-derived Multiple Organism Cloning System), initially established in *Aspergillus niger* (Sarkari et al., 2017) (Figure 2A). GoldenMOCS systems are modular cloning systems designed for fast and efficient construction of plasmids for a variety of purposes, such as constructing complex metabolic pathways (Novak et al., 2020) or genome editing plasmids (Prielhofer et al., 2017). In our approach, this system allows combining three genetic parts into a pMU131 derivative, which can be used to easily exchange promoters, CDSs and terminators.

### FAST-mediated fluorescence at 55°C and 66°C

The GoldenMOCS system was next exploited to generate shuttle plasmids bearing pFAST expression cassettes. The endogenous *pta* promoter from *T. kivui* was first tested to drive the expression of pFAST. The product of the *pta* gene, phosphotransacetylase, is essential for acetate production from acetyl-CoA. Acetyl-CoA is the terminal product of the WLP, which acetogens such as *T. kivui* rely on for biomass and energy generation. For homoacetogens (such as *T. kivui*), excess acetyl-CoA (i.e., not used for biomass generation), is solely used to generate ATP by substrate level phosphorylation, with acetate as the sole product. Because only one *pta* gene could be found in *T. kivui* (Hess et al., 2014), we therefore hypothesized its promoter would be strong and constitutive.

After introducing the resulting plasmid pThermoFAST_01 expressing a codon-optimized pFAST under the control of pPta_Tkv_, fluorescence was assayed with a plate reader using medium to late exponential phase cells. Green or red fluorescence was observed when cells were suspended in phosphate buffer containing ^TF^Lime or ^TF^Coral (Figure 2B). When cells were grown at their optimal temperature (66°C), a relatively weak fluorescent signal was observed corresponding to appr. 9 (^TF^Lime) or 7 (^TF^Coral) times the signal observed for the negative control (thereafter defined as fold change FC_neg_—comparison to cells with the empty backbone plasmid pMU131). We speculated low fluorescence could be due to a potentially high proportion of protein denaturation as growth temperature was close to the melting temperature of pFAST. In addition, the T_m_ determined for the pFAST-^TF^Lime complex is potentially not representative of the T_m_ of pFAST alone, as ligands are susceptible to increase their cognate protein stability (Celej et al., 2003). This potentially beneficial effect of ligand binding on protein thermostability led us to cultivate *T. kivui* in the presence of 20 µM ^TF^Lime at 66°C. This approach however proved unsuccessful, presumably because the fluorogen degraded during the time necessary for growth (data not shown). A second strategy consisted in reducing growth temperature to 55°C. Under this condition, the fluorescence signal significantly rose, with an appr. 43 (^TF^Lime) and 17 (^TF^Coral) FC_neg_.

### A promoter library for *Thermoanaerobacter kivui*

Having established a functional fluorescent reporter system compatible with thermoanaerobic growth conditions, we next expanded the *T. kivui* genetic toolbox by constructing promoter parts compatible with our GoldenMOCS cloning system, which we characterized for their ability to drive pFAST expression. We focused on promoters that were previously used in *Thermoanaerobacter* (*T. kivui* S-layer promoter pSlay_Tkv_, *Thermoanaerobacter* sp. X514 gyrase promoter pGyr_X514_, pMU131 kanamycin resistance promoter pKan_pMU_) (Basen et al., 2018; Jain et al., 2021) and in related bacteria belonging to the *Clostridium* phylogenetic class (*Clostridium acetobutylicum* thiolase promoters pThl_Cac_ and minimal promoter minipThl_Cac_, *Clostridium sporogenes* ferredoxin promoter pFdx_Csp_) (Tummala et al., 1999; Dong et al., 2012; Ng et al., 2013). We also added *Acetobacterium woodii* phosphotransacetylase promoter pPta_Awo_, *T. kivui* glyceraldehyde-3-phosphate dehydrogenase promoter pGap_Tkv_ as well as potentially inducible promoters (*T. kivui* fructose repressor and glycine cleavage system promoters pFruR_Tkv_ and pGcv_Tkv_) and a novel synthetic promoter based on pKan_pMU_ (called pKan_pMU_*), for which the 5′-UTR sequence (comprising the ribosome binding site, RBS) was exchanged with the one from pPta_Tkv_.

After cloning the genetic parts in BB2_pMU131, we measured ^TF^Lime-pFAST fluorescence levels for cells grown at 55°C (Figure 2C). The highest fluorescence was recorded for pPta_Tkv_, which confirmed our initial hypothesis that this promoter allows for strong and constitutive gene expression in *T. kivui*. pSlay_Tkv_ and pGyr_X514_ were confirmed to be also relatively strong, with 54% and 20% of the strength of pPta_Tkv_, respectively.

Interestingly, heterologous promoters from related mesophiles were also shown to be functional in *T. kivui*. In particular, pThl_Cac_ and minipThl_Cac_ exhibited an interesting behavior. Indeed, minipThl_Cac_ mainly differs from pThl_Cac_ by a 84 bp truncation between the transcription start site and the RBS (Dong et al., 2012), which in our case reduced expression by more than half, indicating that the deleted 5′-UTR has a positive effect on expression in *T. kivui*.

For a few promoters, we could not detect any fluorescence (pGap_Tkv_, pPta_Awo_, and pKan_pMU_), which suggests 1) that these promoters are either relatively weak or inactive and 2) that our reporter system was possibly not sensitive enough to quantify low fluorescence levels (supported by a relatively narrow dynamic range, in terms of maximum FC_neg_). By replacing the 5′-UTR sequence from pKan_pMU_ with the 5′-UTR from the pPta_Tkv_ promoter (pKan_pMU_*), we were able to measure low fluorescence levels, indicating that pKan_pMU_ likely drives pFAST expression, albeit at a lower level than pKan_pMU_* (not detectable with our reporter system). Similarly, we could not detect fluorescence for pFruR_Tkv_ and pGcv_Tkv_, with and without supplementing the potential inducers fructose or glycine, respectively (data not shown).

### Plasmid stability and ALE

**FIGURE 3:**
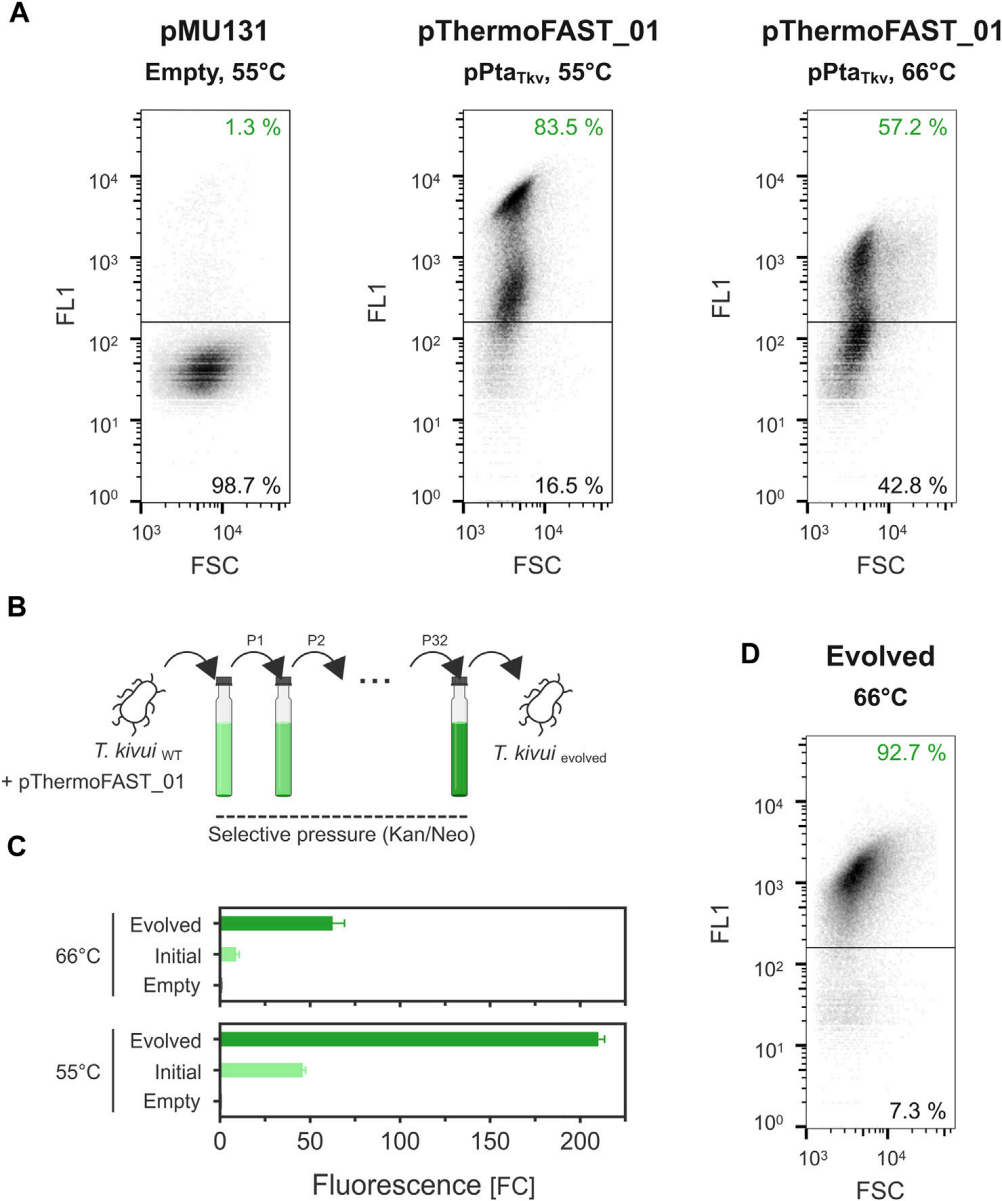
Plasmid instability in *T. kivui* and stabilization by adaptive laboratory evolution. **(A)**. Single-cell fluorescence measured by flow cytometry (density plot, 5 µM ^TF^Lime) for *T. kivui* cells bearing pMU131 (empty backbone, 55°C) and pThermoFAST_01 (pPta_Tkv_, 55°C and 66°C). **(B)**. Adaptive laboratory evolution strategy. **(C)**. Fluorescence measured with a plate reader in the initial and evolved *T. kivui* strains (5 µM ^TF^Lime, *n* = 3, error bars correspond to the SEM). **(D)**. Flow cytometry density plot showing fluorescent cells in the evolved population (66°C, 5 µM ^TF^Lime).

In the previous experiments, the dynamic range of fluorescence at 55°C and 66°C was relatively narrow (up to 43 and 9 FC_neg_, respectively). Plate reader measurements give an output that corresponds to the fluorescence of a cell population, which could potentially be heterogeneous (Mook et al., 2022). Using flow cytometry, we next quantified fluorescence at single-cell resolution and found a significant portion of cells in the *T. kivui* pThermoFAST_01 (pPta_Tkv_) population not to be fluorescent when cultivated at 66°C (43%, Figure 3A), suggesting part of the cell population does not bear or sufficiently express the pFAST gene. Population heterogeneity was also observed at 55°C, in which case both populations exhibited fluorescence (when compared to the empty plasmid population), suggesting both populations expressed pFAST but at different levels. In addition, we compared fluorescence intensity of cells identified as fluorescent (upper panels of Figure 3A) when grown at 55°C and 66°C. We notably observed a stark decrease of fluorescence for the 66°C condition, with a mean of 2,365 relative fluorescence units (rFU) at 55°C compared to 763 rFU at 66°C.

Taken together, the flow cytometry measurements suggested that pMU131 derivatives are not segregationally stable in *T. kivui*, with stability sharply decreasing with increasing temperatures. To enhance plasmid stability, we serially cultivated wild-type *T. kivui* with pThermoFAST_01, maintaining antibiotic pressure along the process, thereby favoring growth of cells that stably and/or strongly expressed genes on the plasmid (such as the antibiotic resistance gene, and the pFAST fluorescent marker) (Figure 3B). This adaptive laboratory evolution approach [ALE (Dragosits and Mattanovich, 2013)] allowed us to obtain a strain with a drastic increase in pFAST expression (Figure 3C). At 55°C, fluorescent signal for the evolved strain was appr. 210 FC_neg_ (≈4.5-fold increase compared to *T. kivui* pThermoFAST_01). Fluorescence increased the most at 66°C (≈7-fold increase compared to *T. kivui* pThermoFAST_01), though the dynamic range was not as high as at 55 °C (FC_neg_ ≈ 63). Flow cytometry analysis further revealed that fluorescence intensity per cell and proportion of fluorescent cells were both increased in the evolved strain at 66°C (mean of 1,230 rFU, 92.7% of the cell population being fluorescent, Figure 3D).

### Plasmid evolution and influence on fluorescence

**FIGURE 4:**
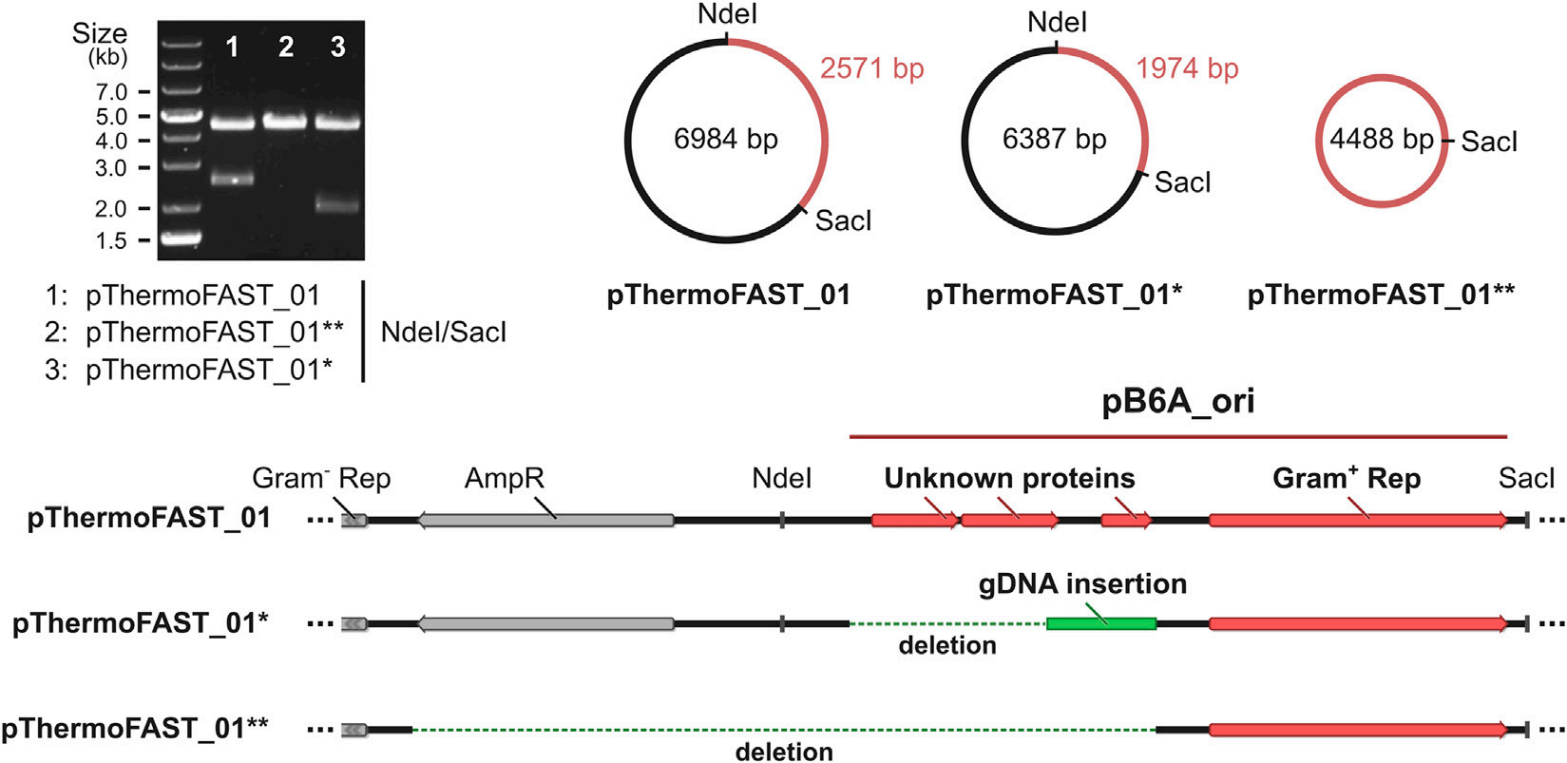
Characterization of the plasmids present in *T. kivui* pThermoFAST_01 after evolution. pThermoFAST_01* and pThermoFAST_01** sequences were obtained by whole plasmid sequencing, and are the product of pThermoFAST_01 evolution.

We hypothesized that the lower population heterogeneity and increased intensity obtained through our ALE approach could be linked with a higher stability of the plasmid at high temperatures, resulting in a higher overall fluorescent signal. To investigate DNA modifications arising at the plasmid level in the evolved strain, we extracted DNA from both the initial and evolved *T. kivui* pThermoFAST_01, amplified the plasmids in *E. coli* using kanamycin as selective pressure, and submitted the resulting vectors for whole plasmid sequencing. While in the initial strain plasmid sequencing showed a single plasmid corresponding precisely to pThermoFAST_01, in the evolved strain sequencing revealed a mixed population of at least two novel plasmids. These two new plasmids display a distinct enzymatic restriction profile on agarose gel electrophoresis, which was used to isolate them in *E. coli*. Coined pThermoFAST_01* and pThermoFAST_01**, both plasmids are shorter in size than pThermoFAST_01 (6,387 and 4,488 bp, respectively, compared to 6,984 bp) (Figure 4). Further sequence analysis revealed both plasmids display a truncation of the Gram-positive origin of replication at the same position, resulting in the deletion of three open reading frames (ORFs) coding for short unknown proteins (96, 108, and 57 amino acid residues), but leaving the *rep* gene intact (likely directly involved in Gram-positive plasmid replication). For pThermoFAST_01*, the 1,029 bp truncation is accompanied by a 432 bp insertion of *T. kivui* gDNA that maps at a single specific locus and contains part of the transposase gene TKV_RS00225. In contrast, pThermoFAST_01** does not contain this insertion and shows an extended deletion that spans along the ampicillin resistance gene (used for *E. coli* plasmid construction) up to the start of the Gram-negative origin of replication. However, upon isolation of pThermoFAST_01* and pThermoFAST_01** and reintroduction into wild-type *T. kivui,* fluorescence levels similar to the original pThermoFAST_01 was observed (data not shown). The increased fluorescence in the evolved strain therefore does not appear to be linked with the deletion of the three ORFs in the Gram-positive origin of replication. Consequently, we further examined whole plasmid sequencing data with respect to identifying additional mutations which might explain the higher fluorescence levels observed for the evolved strain. However, no significant mutation other than the truncation of the Gram-positive origins of replication could be found, indicating that the increased fluorescence displayed by the evolved strain is not a result of plasmid modifications.

## Discussion

The FAST technology enabled simple, direct, quantitative and fast fluorescence measurements in *T. kivui*. To the best of our knowledge, this is the first time a fluorescence reporter system is successfully used in an anaerobic thermophile. Although other fluorescent reporter systems have been developed for other thermophilic microbes, they were only tested or functional in aerobic hosts (Cava et al., 2008; Reeve et al., 2016; Visone et al., 2017; Frenzel et al., 2018; Turkowyd et al., 2020). FAST has so far been demonstrated to be broadly applicable to a wide range of organisms, including mammalian cells, fungi and bacteria (Plamont et al., 2016), and our results with *T. kivui* are therefore likely translatable to other thermophilic anaerobes. Because many thermophilic habitats are naturally low in oxygen or anoxic, the majority of thermophiles are actually strict or facultative anaerobes (Wagner and Wiegel, 2008). For the latter category, FAST could also prove beneficial. In *Geobacillus thermoglucosidasius*, gene expression driven by the pldh promoter has, for example, been shown by an enzymatic assay to be differentially controlled by the redox state, with a complete repression observed in fully aerobic conditions, and high expression at microaerobic and anaerobic conditions (Bartosiak-Jentys et al., 2012). With a single O_2_-independent fluorescent system like pFAST, comparing expression profiles in aerobic, microaerobic or anaerobic conditions therefore becomes easily possible.

Although pFAST could likely be used in other thermophiles growing at temperatures similar or lower than *T. kivui*, its applicability to microbes growing at higher temperatures - such as hyperthermophiles (T_opt_ > 70°C)—might be limited. Indeed, the relatively “low” melting temperature of 69°C likely makes pFAST-^TF^Lime incompatible with hyperthermophilic microbes. The increased thermostability of pFAST over Y-FAST correlates with the previously reported rigidification of the protein structure provided by the introduced mutations (Benaissa et al., 2021). Consequently, specific engineering of thermostability through directed evolution at high temperature could enable the isolation of variants functional in hyperthermophilic conditions.

Increasing pFAST thermostability could also prove beneficial to enhance the overall sensitivity of the system. Even after plasmid evolution through ALE, the bright fluorescence exhibited by the evolved strain was indeed still clearly temperature sensitive, with FC_neg_ values obtained at 55°C much higher than at 66°C (210 vs. 63, respectively). Albeit a relatively narrow dynamic range might not always be problematic, in the present study it restricted the quantification of gene expression to medium/high expression promoters at 55°C. We could therefore only rank promoter strength at 55°C, and whether this ranking is also valid at 66°C requires further investigation. In our hands, it was not possible to detect fluorescence for the pKan_pMU_ construct, despite being reported as functional in various studies (though not in a context where gene overexpression was desired) (Basen et al., 2018; Moon et al., 2019; Le et al., 2020; Dai et al., 2022) and driving the expression of the kanamycin/neomycin resistance gene in pMU131 derivatives. pKan_pMU_ hence likely drives low expression of its cognate gene, and further work is required to precisely quantify its strength.

In contrast to low strength promoters, the pFAST system could easily quantify gene expression driven by seven different promoters, five of which have not been used before in *T. kivui* (pPta_Tkv_, pThl_Cac_, minipThl_Cac_, pFdx_Csp_, pKan_pMU_*). We established the novel pPta_Tkv_ promoter as the strongest one available for *T. kivui*. In *Clostridium ljungdahlii*, the *pta* promoter has been shown to be similarly strong and constitutive (Ueki et al., 2014), which is in stark contrast with the fluorescence levels measured for the *pta* promoter from *A. woodii*. In *A. woodii*, the *pta* gene is organized as a monocistronic unit, whereas in *T. kivui* and *C. ljungdahlii*, *pta* forms an operonic structure with *ackA* (coding for acetate kinase). This differential genetic organization could explain a differential need for high expression, which is supported by transcriptomics data, showing much higher expression levels in *A. woodii* grown in chemostats for the *ackA* gene than for the *pta* gene (Neuendorf et al., 2021).

Flow cytometry analysis using our reporter system suggested that pMU131 derivatives were segregationally unstable, which was severely limiting the fluorescence output at 66°C. Such a phenomenon should normally not arise when maintaining selective pressure. However, antibiotics half-life at higher temperatures is naturally much shorter, which could relieve selective pressure rapidly after early growth. pMU131 replication is driven by the pB6A origin of replication from a natural plasmid occurring in *Thermoanaerobacterium saccharolyticum* (Weimer et al., 1984), a species with a lower optimal growth temperature (60°C), potentially explaining the replicative deficiency observed at higher temperatures. Plasmid segregational stability is regulated by a variety of factors, one crucial being the plasmid copy number (Friehs and Scheper, 2004). Plasmid copy number directly influences the probability that, after division of a plasmid bearing cell, each daughter cell receives at least one plasmid copy. Copy number dictates gene dosage, which in turn determines the expression level of each gene encoded in the plasmid. Based on that principle, our ALE approach aimed at selecting cells with stable, constitutive expression of the antibiotic resistance gene, which could be indirectly monitored by measuring expression of the reporter gene. In the evolved cells, pFAST-mediated fluorescence levels were drastically improved, suggesting gene dosage was increased as intended. Further analysis revealed ALE indeed resulted in the modification of the pFAST-bearing plasmid pThermoFAST_01, and two new plasmid variants with a truncated Gram-positive origin of replication were identified. The deletion results in the removal of about 40% of what was initially identified as the origin of replication, with the elimination of three ORFs coding for hypothetical proteins while maintaining the *rep* CDS and cognate promoter intact. Both evolved plasmids differ from one another by the extent of the deletion, and interestingly by the insertion of part of a transposition element in the longer plasmid variant (ISLre2 family transposase). Because the deletion occurred for both plasmids at the exact same locus, it is possible they derive from one another, in a scenario where, for instance, a transposition element originating from the genome truncated part of the Gram-positive replication origin (yielding pThermoFAST_01*), and its excision later removed the ampicillin resistance gene (yielding pThermoFAST_01**). In both cases the resulting plasmid is shorter and does not bear the three additional transcriptional units present in pThermoFAST_01. Successful transformation of *T. kivui* with either of the two plasmids demonstrates functionality of the truncated variants as novel Gram-positive replication origins. Nevertheless, the increased fluorescence in the evolved strain could not be explained by these alterations, nor by any other occurring at the plasmid level. Therefore, it appears that ALE resulted in additional changes likely occurring in the chromosome that could be responsible for increased pFAST expression. To verify this hypothesis, further studies to unveil the mutations occurring at the genome level after ALE and to understand the mechanisms underlying the enhanced protein expression observed for the evolved *T. kivui* are required. Ultimately, these insights could be of value for metabolic engineering approaches of this industrially relevant microbe.

Despite their advantages for industrial biotechnology, thermophiles in general and thermophilic anaerobes in particular are under-utilized, because many tools required for systems metabolic engineering are far less advanced or altogether lacking compared to their mesophilic counterparts. Facilitating the study and use of thermophiles therefore requires the prior development of genetic tools adapted to their “extreme” lifestyle. In this context, we demonstrated pFAST as a functional fluorescent reporter protein compatible with a strictly anaerobic thermophile and exemplified its use for monitoring gene expression *in vivo* at the population and single-cell level. Additionally, we developed a genetic toolbox for *T. kivui* composed of a modular cloning system, an optimized origin of replication and a library of characterized promoters, which could foster future research in *T. kivui* and related *Thermoanaerobacter*.

## Materials and methods

### *In vitro* thermostability analysis

Determination of melting temperature was performed using the Real-Time PCR System (QuantStudio 5). Proteins [prepared as previously described (Benaissa et al., 2021)] were diluted to 1 μM in PBS buffer and mixed with 10 μM ^TF^Lime. Samples were subjected to thermal ramp from 20°C to 95°C with a ramp rate of 1°C/min during which fluorescence was recorded with the following parameters: Excitation: 470/15 nm and emission: 520/15 nm.

QuantStudio Design and Analysis Software v1.5.2 was used to calculate the T_m_. Melting curves were characterized by two phases: a first phase during which the fluorescence decreases linearly and a second phase where the decrease is more pronounced. The first phase corresponds to the dissociation of the non-covalent protein:fluorogen complex upon increase of temperature. The second sigmoidal phase corresponds to the actual denaturation of the protein. The reported Tm value corresponds to the peak of the first derivative of the fluorescence data, and thus to the inflection point of the sigmoid curve.

### Strains and culture conditions

Strains used in this study are described in Table 1.

**TABLE 1:** Strains and plasmids used in this study.

Strains and DNA	Relevant information	Reference or source
*Strains*		
*T. kivui* LKT-1 DSM 2030	Acetogen, T_opt_ = 66°C	DSMZ
*E. coli* DH10B	Cloning strain	Thermo Scientific
**Golden Gate-compatible parts**		
**Promoters - BbsI donor sites 1, 2**		
BB1_12	empty backbone, BsaI	Sarkari et al., 2017
BB1_pPta_Tkv_	BB1_12 + *T. kivui* pPta promoter	This study
BB1_pKan_pMU_	pKan promoter from pMU131 (synthesis)	This study
BB1_pSlay_Tkv_	*T. kivui* pSlay promoter (synthesis)	This study
BB1_pGyr_X514_	*T.* sp. X514 pGyr promoter (synthesis)	This study
BB1_pPta_Awo_	*A. woodii* pPta promoter (synthesis)	This study
BB1_pKan_pMU_*	pKan promoter from pMU131 with 5′-UTR from *T. kivui* pPta promoter (synthesis)	This study
BB1_pFdx_Csp_	BB1_12 + *C. sporogenes* pFdx promoter	This study
BB1_pThl_Cac_	BB1_12 + *C. acetobutylicum* pThl promoter	This study
BB1_minipThl_Cac_	BB1_12 + *C. acetobutylicum* minipThl promoter	This study
BB1_pGap_Tkv_	*T. kivui* pGap promoter (synthesis)	This study
BB1_pFruR_Tkv_	*T. kivui* pFruR promoter (synthesis)	This study
BB1_pGcv_Tkv_	*T. kivui* pGcv promoter (synthesis)	This study
**CDS - BbsI donor sites 2–3**		
pFAST_Tkv_	pFAST codon optimized for *T. kivui* (synthesis)	This study
**Terminator - BbsI donor sites 3–4**		
tKan_pMU_	pMU131 tKan terminator (synthesis)	This study
***E. coli* - *Thermoanaerobacter* Shuttle Plasmids**		
pMU131	AmpR, ThermoKanR, B6A-RI ori, pUC ori	Shaw et al., 2010
BB2_pMU131	pMU131 engineered for Bbs1 GGA cloning, Recipient Sites 1–4, removable *amilCP*	This study
pThermoFAST_01	BB2_pMU131, pPta_Tkv_, pFAST_Tkv_, tKan_pMU_	This study
pThermoFAST_02	BB2_pMU131, pSlay_Tkv_, pFAST_Tkv_, tKan_pMU_	This study
pThermoFAST_03	BB2_pMU131, pGyr_Tkv_, pFAST_Tkv_, tKan_pMU_	This study
pThermoFAST_04	BB2_pMU131, pThl_Cac_, pFAST_Tkv_, tKan_pMU_	This study
pThermoFAST_05	BB2_pMU131, minipThl_Cac_, pFAST_Tkv_, tKan_pMU_	This study
pThermoFAST_06	BB2_pMU131, pFdx_Csp_, pFAST_Tkv_, tKan_pMU_	This study
pThermoFAST_07	BB2_pMU131, pKan_pMU_*, pFAST_Tkv_, tKan_pMU_	This study
pThermoFAST_08	BB2_pMU131, pKan_pMU_, pFAST_Tkv_, tKan_pMU_	This study
pThermoFAST_09	BB2_pMU131, pPta_Awo_, pFAST_Tkv_, tKan_pMU_	This study
pThermoFAST_10	BB2_pMU131, pGap_Tkv_, pFAST_Tkv_, tKan_pMU_	This study
pThermoFAST_11	BB2_pMU131, pFruR_Tkv_, pFAST_Tkv_, tKan_pMU_	This study
pThermoFAST_12	BB2_pMU131, pGcv_Tkv_, pFAST_Tkv_, tKan_pMU_	This study
pThermoFAST_01*	Evolved pThermoFAST_01, short plasmid	This study
pThermoFAST_01**	Evolved pThermoFAST_01, long plasmid	This study

*E. coli* was grown in LB medium (10 g L^−1^ tryptone, 10 g L^−1^ NaCl, 5 g L^−1^ yeast extract) at 30°C, with carbenicillin (100 mg L^−1^) or kanamycin (50 mg L^−1^) as needed. For solid media, 15 g L^−1^ agar was added.

*T. kivui* LKT-1 DSM 2030 was handled and maintained anaerobically as previously described (Hess et al., 2014), with some modifications. All experiments were conducted in a vinyl anaerobic chamber (Coy lab, Grass Lake, United States) or in an anaerobic workstation (Concept 500, Baker Ruskinn, Bridgend, UK). Mineral medium was used throughout the study with the following composition, per liter: 5.5 g glucose monohydrate, 7.8 g Na_2_HPO_4_*2H_2_O, 6.9 g NaH_2_PO_4_*H_2_O, 210 mg K_2_HPO_4_, 160 mg KH_2_PO_4_, 250 mg NH_4_Cl, 225 mg (NH4)_2_SO_4_, 438 mg NaCl, 91 mg MgSO_4_*7H_2_O, 6 mg CaCl_2_*2H_2_O, 2 mg FeSO_4_*7H_2_O, 5.41 g KHCO_3_, 500 mg Cysteine-HCl*H_2_O and 10 mL DSM141 modified Wolin’s mineral solution. 2 g L^−1^ yeast extract was only added for transformation experiments and plates. Plates were made using the same medium supplemented with either 8 g L^−1^ Noble agar (Carl Roth, Karlsruhe, Germany) or 3 g L^−1^ Gelrite (Carl Roth). 200 mg L^−1^ kanamycin or neomycin were added when required. Hungate tubes or 125 mL serum bottles were used for anaerobic cultivation. Prior to cultivation, the headspace was flushed with a gas mixture containing 20% CO_2_ and 80% N_2_, and the pressure set to 2 bar. A rotary shaking incubator (55°C) or a shaking water bath (66°C) were used for cultivation.

### Construction of a golden gate-compatible shuttle and pFAST plasmids

Plasmids used in this study are described in Table 1. DNA parts used in this study are further described in Supplementary File S1.

Site directed mutagenesis was first performed to remove a BbsI restriction site in the plasmid. A Golden Gate cassette containing BbsI recipient sites 1 and 4 was next inserted by enzymatic restriction between SacI and KpnI sites, yielding BB2_pMU131.

Next, Golden Gate assembly (GGA) was used to create pFAST expression plasmids. Promoters were first synthesized (Twist Bioscience) or cloned (PCR and BsaI GGA in BB1_12) in plasmids containing BbsI donor sites 1 and 2. pFAST was codon optimized for *T. kivui* and synthesized (Twist Bioscience) in a plasmid containing BbsI donor sites 2 and 3. The Kan terminator from pMU131 *thermoKanR* gene was synthesized (Twist Bioscience) in a plasmid containing BbsI donor sites 3 and 4. Promoters, pFAST and tKan parts were subsequently combined by GGA in BB2_pMU131 using BbsI-HF (NEB), yielding the pThermoFAST plasmid suite.

### Transformation of *Thermoanaerobacter kivui*

Plasmids were introduced in *T. kivui* by natural competency as previously described (Basen et al., 2018). Briefly, cells were cultivated overnight at 66°C in 2 mL mineral medium supplemented with 2 g L^−1^ yeast extract and 1 µg of plasmid. Cells were then embedded in hot selective medium at various dilutions and left under anoxic conditions until plates solidified. Neomycin was preferred over kanamycin for initial selection. Plates were incubated until colony formation at 66°C in a custom anaerobic jar under 2 bar N_2_:CO_2_ (80:20). Plasmid presence was assessed by PCR targeting the pFAST expression cassettes using colonies grown in liquid cultures as DNA templates.

### Fluorescence assays in plate reader

To measure expression using pFAST as a reporter, a plate reader photometer was routinely used (Infinite M200, Tecan, Männedorf, Switzerland). Cells bearing the pFAST plasmids were cultivated in 3 mL mineral medium at 55°C or 66°C until mid-late log phase (0.8–2.0 OD_600_ units). Cells were concentrated by centrifugation to 5 OD_600_ units in 70 mM sodium phosphate buffer pH 7.4 with 5 µM fluorogen (^TF^Lime or ^TF^Coral, Twinkle Factory, Paris, France). Fluorescence of 100 µL of cell suspension was quantified at 30°C in relative fluorescence units (^TF^Lime: excitation 485/20 nm, emission 535/25 nm, gain 50 and 60; ^TF^Coral: excitation 485/20 nm, emission 595/35 nm, gain 75). Fluorescence of a well containing 100 µL of the fluorogen solution without cells was subtracted to the values obtained. Results were normalized using the fluorescence value obtained for cells bearing pMU131 (no insert control).

### Flow cytometry

Single-cell fluorescence was measured using flow cytometry (Cube 8, Sysmex, Kobe, Japan). ^TF^Lime-pFAST fluorescence was monitored using a blue solid-state laser (488 nm) for excitation and a green filter (536/40 nm) for emission. Cells were grown to ≈1 OD_600_ unit and 5–10 µL were mixed with 1 mL H_2_O containing 5 µM ^TF^Lime. At least 50,000 events were analyzed for each sample. Analysis was performed using FlowJo (v10.8.2).

### Whole plasmid sequencing

DNA was extracted from overnight cultures of *T. kivui* (Hi Yield Mini Plasmid DNA extraction kit, SLG) and directly introduced into *E. coli*. Cells were selected with kanamycin in liquid and plasmid DNA was purified, and sent for whole plasmid sequencing (Plasmidsaurus). The resulting files were analyzed using Geneious Prime 2023.1.1 (https://www.geneious.com).

## Data Availability

The original contributions presented in the study are included in the article/Supplementary Material, further inquiries can be directed to the corresponding author.
