# Supplementary material for: A fluorescent reporter system for anaerobic thermophiles
Source: Front Bioeng Biotechnol. 2023 Jul 5;11:1226889. doi: 10.3389/fbioe.2023.1226889 (PMC10355840; doi:10.3389/fbioe.2023.1226889)
Supplement: Supplementary file 1 [file Presentation1.PPTX]

## Slide 1
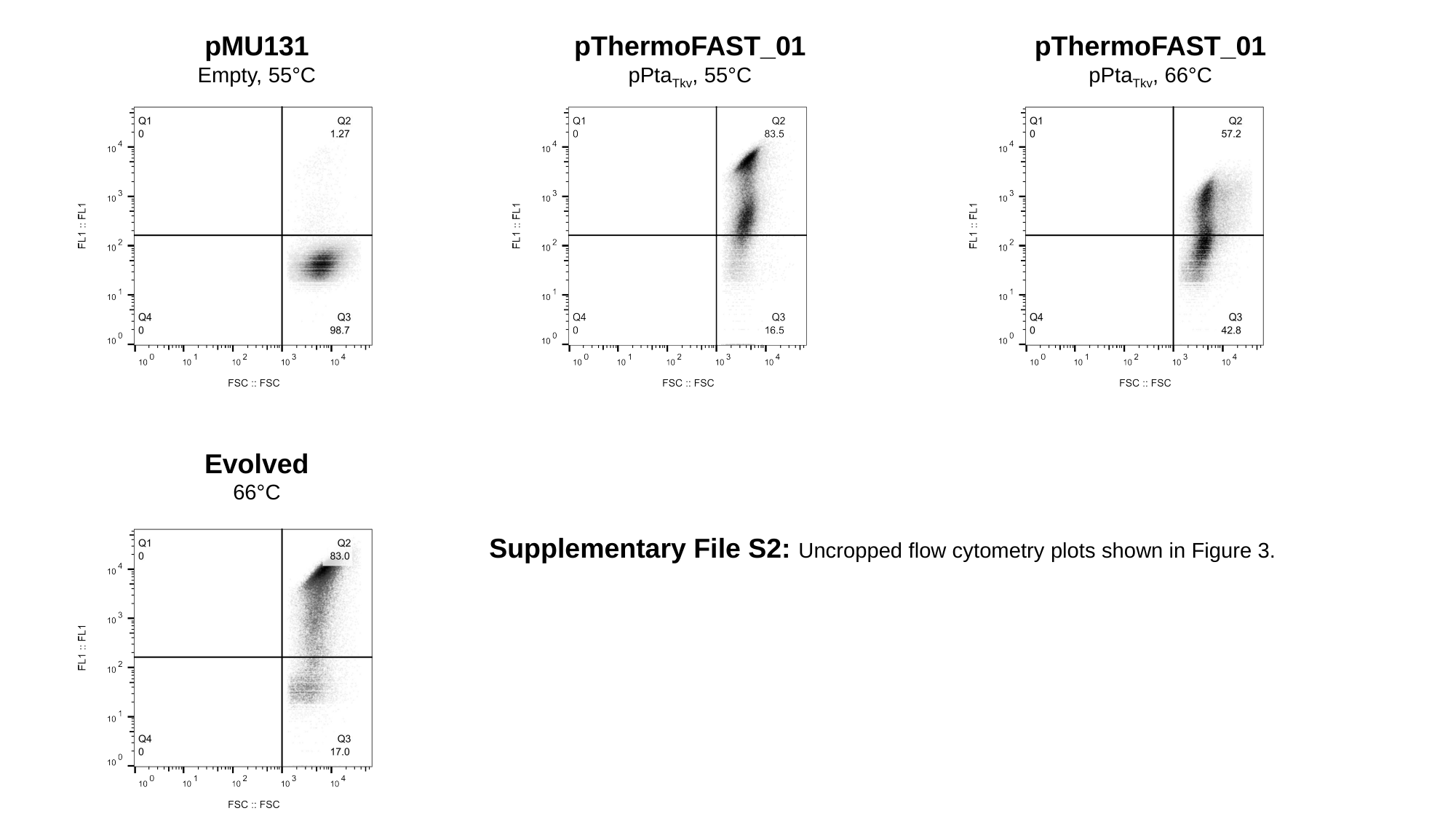

pThermoFAST_01
pPtaTkv, 66°C
pMU131
Empty, 55°C
pThermoFAST_01
pPtaTkv, 55°C
Evolved
66°C
Supplementary File S2: Uncropped flow cytometry plots shown in Figure 3.
