# Supplementary material for: A fluorescent reporter system for anaerobic thermophiles
Source: Front Bioeng Biotechnol. 2023 Jul 5;11:1226889. doi: 10.3389/fbioe.2023.1226889 (PMC10355840; doi:10.3389/fbioe.2023.1226889)
Supplement: Supplementary file 2 [file DataSheet1.DOCX]

A fluorescence reporter system for anaerobic thermophiles

Rémi Hocq, Sara Bottone, Arnaud Gautier and Stefan Pflügl

**Supplementary file S1: *Thermoanaerobacter kivui* genetic parts**

Fusion sites are underlined, Bbs1 recognition sites are in bold.

**Shuttle plasmid: pMU131 cloning site with GGA receiving sites 1 and 4**

>BB2_Cloning_site_1_4_amilCP

GAGCTCGGTCTCCGATCGGAGGC**GTCTTC**GGATCCAGAATTCGTGATACCTGCGCGCGGAGGGAGTTTACGGCTAGCTCAGTCCTAGGTACTATTACTAGTGAAAGAGGAGAAATACTACATGAGTGTGATCGCTAAACAAATGACCTACAAGGTTTATATGTCAGGCACGGTCAATGGACACTACTTTGAGGTCGAAGGCGATGGAAAAGGTAAGCCCTACGAGGGGGAGCAGACGGTAAAGCTCACTGTCACCAAGGGCGGACCTCTGCCATTTGCTTGGGATATTTTATCACCACAGTGTCAGTACGGAAGCATACCATTCACCAAGTACCCTGAGGACATCCCTGACTATGTAAAGCAGTCATTCCCGGAGGGCTATACATGGGAGAGGATCATGAACTTTGAAGATGGTGCAGTGTGTACTGTCAGCAATGATTCCAGCATCCAAGGCAACTGTTTCATCTACCATGTCAAGTTCTCTGGTTTGAACTTTCCTCCCAATGGACCTGTCATGCAGAAAAAGACACAGGGCTGGGAACCCAACACTGAGCGTCTCTTTGCACGAGATGGAATGCTGCTAGGAAACAACTTTATGGCTCTGAAGTTAGAAGGAGGCGGTCACTATTTGTGTGAATTTAAAACTACTTACAAGGCAAAGAAGCCTGTGAAGATGCCAGGGTATCACTATGTTGACCGCAAACTGGATGTAACCAATCACAACAAGGATTACACTTCGGTTGAGCAGTGTGAAATTTCCATTGCACGCAAACCTGTGGTCGCCTAATAAGCTTAAAAAAAAACCCCGCTTCGGCGGGGTTTTTTTTTCGCTGCTTGCGCGCAGGTATCTGAATTCGTCGACAAGCTTCTCGAG**GAAGAC**GCCGCTCCGGGGAGACCAGGTACC

**Promoters: donor sites 1 and 2**

>pPta_Tkv_

**GAAGAC**GCGGAGCTTCCATTACCCTTTCAAAAAATTTTTAAGATGTACTTACTATTTTATATAAAATATGATAAAATGTAAAATAGGAACTGTGTATACAATATATTTGGTAAATATTTAGGTAGAAAGGAGATAGTAACATGGC**GTCTTC**

>pKan_pMU_

**GAAGAC**GCGGAGGAAAACAACTTTGAAAAAGCTGTTTTCTGGTATTTAAGGTTTTAGAATGCAAGGAACAGTGAATTGGAGTTCGTCTTGTTATAATTAGCTTCTTGGGGTATCTTTAAATACTGTAGAAAAGAGGAAGGAAATAATACATGGC**GTCTTC**

>pSlay_Tkv_

**GAAGAC**GCGGAGAGCTACTCATCCCAGATTCAAAAAAATACTCAAAGAAATGTTAAATTAATATTACAGAAATATTACAAATTGGTAACAATTATTGACTCATTAAATACCTGATGCTATAATATAATCAGGTTGTTTATCCAAAACATACTACATACTCTAAAATGTACTTACATAGGTAAAAAATTTTTGAAAGGCTTATGCCTTTCAAAACAATAATATAAAAATTAAAATACAAATACAAGGAGGAGGATTGACTGCATGGC**GTCTTC**

>pGyr_X514_

**GAAGAC**GCGGAGTTTCCTATTTTTACTCTTCTTTCTGGTTAAAGCAATACTACATTTCCTTCTTCATCATCTACTCCTAAAACTTCGGATACGAAGTCAGCAATTTGTATATTCCTATGTCAAGTTTTAAAAAATCTTCAGAGGTAGCCATTGAATTCCCTCCAATTTAAGTATATTATATTATAGGTACATGTGTTAAGTATTATTATATTATAGGTAATAATAAAAATAAAATTATCTCATTTCCCGGGAAAATTTTTCGGGAAATATTTTGAATTGAGCAATTTTTAAAATAATGATAAAATAAAACTGTTGGATAAATTTGCGGAGGTTGTGAATCATGGC**GTCTTC**

>pPta_Awo_

**GAAGAC**GCGGAGCCTTTTTAAAAGGACAATTTATAAAACATCCCCATTATTTGGTCAACTTGATCGGTTCTGCGCTGGAATTGATTAAGTTGACTCTATTTTTTTCTTAACAAATAAAATTGCTTGCCATCATTAAATTACCTTCTTTTTAATGATGGCAAGTAATTGATATGAATTGATAATAAATTATATGTAACAAAATTGTTTCTTTATTATACGTTTGAATTAGTGTATAATGTTTTATATGAAAATGAGGTTAACATGGC**GTCTTC**

>pKan_pMU*_

**GAAGAC**GCGGAGGAAAACAACTTTGAAAAAGCTGTTTTCTGGTATTTAAGGTTTTAGAATGCAAGGAACAGTGAATTGGAGTTCGTCTTGTTATAATTAGCTTCTTGAACTGTGTATACAATATATTTGGTAAATATTTAGGTAGAAAGGAGATAGTAACATGGC**GTCTTC**

>pFdx_Csp_

**GAAGAC**GCGGAGGTGTAGTAGCCTGTGAAATAAGTAAGGAAAAAAAAGAAGTAAGTGTTATATATGATGATTATTTTGTAGATGTAGATAGGATAATAGAATCCATAGAAAATATAGGTTATACAGTTATATAAAAATTACTTTAAAAATTAATAAAAACATGGTAAAATATAAATCGTATAAAGTTGTGTAATTTTTAAGGAGGTGTGTTACATGGC**GTCTTC**

>pThl_Cac_

**GAAGAC**GCGGAGTTTTTAACAAAATATATTGATAAAAATAATAATAGTGGGTATAATTAAGTTGTTAGAGAAAACGTATAAATTAGGGATAAACTATGGAACTTATGAAATAGATTGAAATGGTTTATCTGTTACCCCGTATCAAAATTTAGGAGGTTAGTTCATGGC**GTCTTC**

>minipThl_Cac_

**GAAGAC**GCGGAGTATATTGATAAAAATAATAATAGTGGGTATAATTAAGTTGTTAGGAGGTTAGTTAGACATGGC**GTCTTC**

>pGap_Tkv_

**GAAGAC**GCGGAGGTGACTGATGAAGGAGCTGCCTTTGAAATTTTAAAAATTGTTTAATGCACCTTTTGAGGTGTTTATAAAATAAAAATATTGTTTTAAGGAGGAATATCACATGGC**GTCTTC**

>pFruR_Tkv_

**GAAGAC**GCGGAGTATTCAACTATAAAGAGTCCTCCTCCTTGGGGGACTCTTTTATTTGCTTTAAAACAGGCATTGCCTGTTTTATTTTTTTGCCTATTGACAAAGTAAAAATGATAATATATAATCAAAATTGAGCAAAATCATTCACAATTAAGCAGGACAAAATTGAATGGATGTGCATGGC**GTCTTC**

>pGcv_Tkv_

**GAAGAC**GCGGAGCCATAATCTATTTTATCACAGGGGTTGTCTCTTGTTTTTTATCACTTAAAAAATCCTACGATAATTTTACACTATGGATGAAGATAGCGAGAGATATCTTTTTTAAAAAGATAGCCGAAGGGGAAATATAAAGGCCCGCCAAGCCTTTATAAAAGCTCTCAGGCAAAAGTATCGCTATCGGATAGACCTCTGGAAAGTCTCGTATAGAGCGCCGAAGGAGCAATACTAAGTAGAAGCTCTCAGGTCAAAAAACAGGGGAGTTATGATGGCATTTGTTTTGTTGTGCTCATCATAACTCCTCTTTTAAAATTTTATGAAAGGGTGTGATGTCTCATGGC**GTCTTC**

**CDS: donor sites 2 and 3**

>pFAST_Tkv_

**GAAGAC**GCCATGGAACATGTTGCATTTGGTTCAGAAGATATTGAAAATACTCTAGCAAATATGGACGATGAACAACTTGATCGGTTAGCTTTTGGTGTTATTCAGTTAGATGGTGATGGTAATATACTTCTTTATAATGCGGCAGAAGGGGACATTACTGGGCGAGATCCTAAACAAGTCATTGGAAAAAATTTCTTTAAAGATGTCGCGCCTGGAACAGATACTCCTGAATTTTATGGTAAATTTAAGGAAGGTGCTGCCAGCGGAAACCTTAATACGATGTTTGAATGGACTATACCTACATCACGCGGACCAACAAAAGTTAAAGTGCATTTAAAAAAAGCATTGTCAGGTGATAGGTATTGGGTGTTTGTAAAACGCGTGTAAGCTTGC**GTCTTC**

**Terminator: donor sites 3 and 4**

>tKan_pMU_

**GAAGAC**GCGCTTTACCTAGATTTAGATGTCTAAAAAGCTTGGCGTAATCATGCGCTGC**GTCTTC**

**pMU131 Gram-positive origin of replication, evolved**

>pMU131_ori_evolved

CTTGATATATTAGAAAAAAGCGTACTCACGAAGTAAGAATTTGTAAAAAAAGAAGGGGGGATTTTTTTGGATGAGAGTTTGTACAAGCAGATTTTAAGTAATATTATTATTACTCGTGATTATTGTAAAAATGTTTTAGATAATATAAAGTTCAATGAAAAAATAATTGATTATTATGTTATGTTACAAAATGATGTTTTTATTGATTTTACTAATAAAATAAATTCAATAAGGGATTGTAATAAATATTGGTATTTGGATGTTTATAAAAAGCAGAAAATAAAGGATTTTAAAAAGACTAATTTGTGTAAAGATAAGTTCTGTAATAATTGTAAGAAAGTTAAACAGGCTTCAAGAATGCAAAAATATATTCCTGAATTACAGAAATACAAAGATGGCTTATATCATTTTATATTTACTGTTGAAAATGTGCCAGGTAGTGAATTAAGAGATACTATTGATAGGTTGTTTAAGTCTTTTAAGTCATTTACAAGGTATTTAAGTGGTAATCTTAAAATAAAAGGTGTTAATTTTGATAAATGGGGTTATAAAGGCTGTGTAAGGTCTTTAGAGGTAACTTATAGTATGATTGATAATCATATTATGTATCATCCACACTTGCATGTTGCGATGATATTAGATCCTTTTTACGATGGTTTTAATGTTGAAAGGATGCATATAATTAATAAGTTTAGTTATAGCTATGGTGTTTTAAAAAGGTTGTTTACTGATGATGAATTATTAATTCAAAAAATTTGGTATTTATTGTTTAATAATATTGAGGTTAACATGGCCAATATAAATAATTTAGAGGATGGTTATTCTTGTTTAGTTAATAAGTTTAGTGATTATGATTATGCGGAGCTGTTTAAGTATATTTGTAAAAATACTGATGAACAAGGTTTACTTATGACTTATGATATTTTTAAAGATTTATATTTTGCATTACATAATGTTCATCAGATACAAGGCTATGGTTGTTTATATAATATAAGAGATGATACTCAATTAGATTTAAAGGTTGATGACATTTATAATGATTTGATTGATTTATTACAAGTTACAGAAAATCCTATACAGTCTATGGAAACTGTACAGGATTTATTAAAGGATACTGAATATACAATAATAAGCCGTAAGCGTATATTTAAGTATCTAACACAATTATATCATAAGGATTGATATTTATACCGTCTGTCGGACTCATGCGGAGGGGGACTTGAGGGGGTCTCCCCTCGCATTGTACGACAGACGGTATTATTATTATACAAATTTTTTTTATGTAATTTTTTTTGTGTAATTTTTTTATACAAATAATATT
